# The Social Evil—Its Prevalence and Results

**Published:** 1911-04

**Authors:** R. R. Kime


### THE SOCIAL EVIL—ITS PREVALENCE AND RESULTS.

By R. R. Kime, M. D.

Few of the general public, and many physicians, do not really recognize the prevalence of venereal diseases and their serious results physically and morally on mankind.

To the student of sociology and especially medical sociology, it is a serious problem, fraught with serious results and demands serious consideration.

As the medical profession learns more of the immediate and remote results of venereal diseases, then we recognize more fully its devastating influence on mankind.

When we recognize that syphilis affects the skin, the mucous membranes, bones, teeth, liver, nervous system, brain, spinal cord, and is transmitted not only from man to woman, but to the offspring with marks of the disease often worse than the disease itself; that gonorrhoea not only affects the urethra and vagina, but all the generative organs in both male and female, often rendering them both sterile. In the female, it produces serious diseases of the tubes, ovaries, pelvic peritoneum, frequently resulting in death or serious abdominal operations to save life, that the gonococcus often causes serious disease of eyes and blindness of the newborn, that gonorrhoea secondarily produce rheumatism, disease of the joints, of the pleura, of the heart, eac., then we begin to recognize that "gonorrhoea is far more serious than a bad cold," and that the possessor of either syphilis or gonorrhoea is a walking pestilence to be dreaded and avoided.

We quarantine and restrict smallpox, scarlet fever and diphtheria, and require registration of typhoid fever and tuberculosis. while the sypthlitic and gonorrhoeic go unrestrained to spread the disease like a pestilence in darkness. When will common sense and justice prevail and innocent victims be saved from this physical and moral curse? We need an awakening in venereal diseases like that against tuberculosis to check the progress of the "great black plague" that stalks in darkness and secrecy. Light kills the germ of the "great white plague" so will enlightenment kill the germs of the "great black plague." Let every citizen banish prudery, exercise common sense, and turn on the light.

Warbarse, in Medical Sociology, tells us that in these United States there are "800,000 males reaching maturity each year, 500,000 of these have gonorrhoea. Of 14,000,000 male adults under thirty 8,000,000 have gonorrhoea or its sequellae. 75% of operations for pelvic inflammation in the female are due to gonorrhoea. 60 to 75% of the women who marry accept the risk of contracting venereal disease from their husbands. 75% of enforced childless marriages are due to gonorrhoea—30% in the male and 45% in the female. Gonorrhoea is one of the most prolific cause of extra-uterine pregnancy. 73% of the spontaneous abortions due to endometritus are caused by venereal disease. There are 110,000 blind persons in the United States, mostly due to gonorrhoea. 80% of blindness in newborn is due to gonorrhoea. No statistics of syphilis in the U. S. Pinaud states, "of ten thousand consecutive cases of miscarriage in Europe 42% were associated with syphilis." 20,000 children die annually of syphilis in France. In the U. S. army syphilis causes the discharge of more men for physical disability than tuberculosis."

Dr. Kelly, of Johns Hopkins hospital, states—"In Baltimore, that gentle, moral city, in my own clinicat the Johns Hopkins alone, we have had over 189 cases of little children, some but wee babes in arms, violated, and in every instance, infected with the most disgusting diseases to which flesh is heir, gonorrhoea or syphilis, and in a number of instances the poor little innocent sufferer had contracted both in addition to her defloration. Dr. Pollock, who conducts this clinic, after a searching examination of our own and other local records, has estimated conservatively that from 800 to 1,000 children between the ages of one and fifteen are yearly inoculated in our city alone on the altar of perverted brutal male lust, made to suffer physical tortures, defloration, and often infected with these frightful disease from which they may never recover."

Dr. Kelly further states—"Most cases of locomotor-ataxia are due to syphilis. Were it not for syphilis and alcoholism with their immediate and remote results, our insane asylums would in a large measure, be depopulated. That almost all the cases of paralysis in men under forty are due to syphilis. The Wassermann reaction shows that almost all prostitutes have syphilis. New York is estimated to have 200,000 syphilitic subjects, 40 to 50,000 prostitutes, and from one hospital 3,000 cases of venereal diseases were reported in one year."

Judge Ben Lindsay said—"Conditions in Denver were not exceptional, the trail of the beast is to be found in your city and in mine as well as in Colorado."

Lydston, in his Diseases of Society, P. 113, says, "In St. Petersburg 83% of the prostitutes have syphilis. In Stuttgart every prostitute becomes infected with gonorrhoea at least once a year. In Berlin 50% of the loose women have gonorrhoea constantly and the rest frequently. It is estimated that there are 150,000 syphilitics in Berlin. Paris has a still greater proportion."

Dr. Morrow says: "New York has 200,000 syphilitcs" and Lydston further states that "Chicago rivals New York, and that one-fourth the adult population of our large cities is affected by venereal disease in one form or another or by their sequellae."

Morrow—Social Disease and Marriage," Page 348—"The public does not know that these diseases are often conveyed in the sacredness and what should be the safeguard of the marriage relation, that they embrace among their victims a vast number of virtuous wives and innocent children, that the chief sufferers are by no means the greatest offenders against morality. We cannot impute to divine agency a disease which, like syphilis, ruthlessly smites the innocent wife and her offspring; we cannot consider a disease like gonorhoea a merited punishment when conveyed under relations which society has sanctioned as lawful, honorable and virtuous—a disease which ruins her health, extinguishes her exceptional capacity, and condemns her to a life of invalidism or to mutilation at the hands of the surgeon to save her life. The public does not appreciate the tact that the immense majority of the victims of venereal diseases are the young, the inexperience, and the irresponsible, through ignorance."

So we might extend and multiply these statements but the picture is black enough and when you recognize that these same conditions exist in Atlanta, then I feel that it is high time that you as ministers were waking up to the condition as it exists.

If you desire to save men's souls they must be enlightened as to the pitfalls and dangers that beset their pathway. They must be taught proper moral standards for control of the physical body and passions. They should be taught a single moral standard, not by lowering woman, but by raising man's standard and in so doing elevate woman to a yet higher standard, for most women fall because of the sins of men.

It is not alone the duty of the medical profession to impart this instruction, but the clergy also should inform themselves on this subject and lend a helping hand. All professions and all intelligent men and women should aid in the dissemination of knowledge and in the inoculation of the right moral standards that will tend to check the progress of the social vices. We ask and urge one and all to join hands with us in the discussion and study of these social vices that we may all be the better able to understand their propagation, dissemination, and control. You, as ministers, cannot afford to pass this subject by in dignified silence while we, as physicians, treat the men of your congregations for venereal diseases and their complications, and later treat their wives for the same disease innocently contracted and tell them white lies to deceive them as to their true condition. Such methods have been tried long enough to prove their inadequacy and worthlessness. The results are that venereal diseases to-day are more prevalent than tuberculosis and almost as deleterious in results.

We urge you to action in a crusade against venereal diseases similar to that against tuberculosis, a crusade based upon proper knowledge and conduct with discrimination and judgment,

To do this come and let us reason together that we may be properly prepared for our individual duties and responsibilities in this work.

Warbasse.—Med. Sociology.
Lydston.—Diseases of Society.
Morrow.—Social Diseases and Marriage.
Robinson.—Never Told Tales.

